# Acute hemodynamic effects of adaptive servoventilation in patients with pre-capillary and post-capillary pulmonary hypertension

**DOI:** 10.1186/s12931-015-0298-z

**Published:** 2015-11-04

**Authors:** Karen M. Olsson, Anika Frank, Jan Fuge, Tobias Welte, Marius M. Hoeper, Thomas Bitter

**Affiliations:** Department of Respiratory Medicine and German Center of Lung Research (DZL), Hannover Medical School, 30623 Hannover, Germany; Heart and Diabetes Center NRW, Ruhr-University Bochum, Bad Oeynhausen, Germany

**Keywords:** Hypertension, Pulmonary, HFpEF, Hemodynamics, Servoventilation, Noninvasive ventilation

## Abstract

**Rationale:**

The hemodynamic effects of adaptive servoventilation (ASV) in patients with pulmonary hypertension (PH) are unknown.

**Methods:**

A series of clinically stable patients with pre- or post-capillary PH underwent ASV therapy (endexpiratory positive airway pressure support 12–14 cm H_2_O, pressure support 4–10 cm H_2_O) during right heart catheterization. Hemodynamics were measured at rest, at the end of a 15-min episode of ASV therapy, and 15 min after ASV completion. Hemodynamic variables included heart rate, blood pressure, right atrial pressure (RAP), mean pulmonary artery pressure (PAPm), pulmonary arterial wedge pressure (PAWP), cardiac output and pulmonary vascular resistance (PVR).

**Results:**

The study enrolled 33 patients; 12 patients with post-capillary PH due to heart failure with preserved ejection fraction, and 21 patients with pre-capillary PH due to pulmonary arterial hypertension (*n* = 8) or chronic thromboembolic pulmonary hypertension (*n* = 13). ASV was well tolerated by all patients and resulted in reductions in systolic blood pressure (−8 mmHg, *p* = 0.01), PAPm (−5 mmHg, p <0.001) and PVR (−10 %, *p* = 0.01). Right and left filling pressure increased, while the cardiac output decreased (−0.4 L/min; *p* < 0.001). The hemodynamic effects of ASV were similar in both patient populations.

**Conclusions:**

ASV had moderate hemodynamic effects in patients with PH of various origins, most importantly a decline in systolic blood pressure, PAPm and cardiac output. ASV was safe and well tolerated during this short-term study, but the observed drop in blood pressure and cardiac output may be of concern if ASV is applied in patients with advanced PH and severely impaired right ventricular function.

## Introduction

Pulmonary hypertension (PH) is defined by a mean pulmonary artery pressure (PAPm) ≥25 mmHg at rest. Depending on the level of the pulmonary artery wedge pressure (PAWP), PH is divided into pre-capillary and post-capillary forms, the former encountered in patients with pulmonary arterial hypertension (PAH), chronic thromboembolic pulmonary hypertension (CTEPH), or PH due to lung disease; the latter in patients with left heart disease such as heart failure with preserved ejection fraction (HFpEF) [1, 2].

Although some forms of PH may occur in young patients, the majority of patients with PH is in an advanced age and often presents with co-morbidities such lung disease, left heart disease, obesity and sleep-related breathing disorders. Obstructive or central sleep apnea has been found in about one fourth of patients with pre-capillary PH [3]. In addition, hypocapnic respiratory failure and nocturnal period breathing are occasionally found in patients with PH [4, 5]. Moreover, these patients are at risk of developing acute respiratory failure due to decompensation of their underlying illness or due to intercurrent diseases, such as pneumonia. Hence, in some of these patients, noninvasive ventilation (NIV) may be applied, either as acute or long-term therapeutic intervention.

In patients with chronic left heart failure application of continuous positive airway pressure (CPAP) or NIV reduced pulmonary congestion and left ventricular preload by increasing intrathoracic pressure and by reducing afterload via reduction of transmural wall pressure. Cardiac index remained stable or increased [6–8]. One form of NIV is adaptive servoventilation (ASV), an automatic bi-level positive airway pressure therapy with anticyclical adaption of pressure support [9]. In patients with chronic left heart failure, ASV did suppress nocturnal apneic and hypopneic events and had a positive impact on cardiopulmonary function and cardiac remodeling [9–11]. However, the patients included in these studies did not suffer from severe PH and it is possible that the administration of positive pressure breathing could have deleterious hemodynamic effects in such patients. Therefore, we evaluated the acute hemodynamic effects of NIV in patients with pre-capillary or post-capillary PH during right heart catheterization.

## Methods

### Patients

Patients admitted to our hospital for diagnostic work-up or follow-up assessment of pulmonary hypertension were invited to participate in this study. Patients with PAH, CTEPH and PH-HFpEF according to current diagnostic criteria were eligible. Patients with PH due to lung disease were excluded as were patients already receiving noninvasive ventilation for other reasons.

All patients provided written informed consent and the study was approved by the local ethics committee of Hannover Medical School institutional review board (Ethikkommission Medizinische Hochschule Hannover).

### Right heart catheterization

Right heart catheterizations were performed via a jugular approach following a standardized protocol. The pressure transducer was zeroed at the mid-thoracic level and pressure readings were done at end-expiration [2]. Measurement included right atrial pressure, PAPm, PAWP and mixed venous oxygen saturation. Cardiac output was measured by thermodilution with the reported value being the average of at least three recordings with less than 10 % variation. Pulmonary vascular resistance was calculated according to standard formula. Baseline measurements were made after a 15 min resting period following catheter insertion. Intervention measurements were made at the end of a 15-min ASV period, and post-intervention (recovery) measurements were made after another 15 min of rest and unsupported breathing.

### Capillary and mixed-venous blood gas analyses

Experienced technicians obtained arterialized capillary blood gases from earlobes. Mixed-venous blood was obtained from the pulmonary arteries during right heart catheterization. The blood samples were analyzed without delay using a standard device (Radiometer, Copenhagen).

### Adaptive servo ventilation

After individual mask fitting, ASV was initiated during a 15 min period using PaceWave™ modus with a standardized protocol of pressure support level titration under continuous blood pressure monitoring (Carescape V100). Starting at 4 cm H_2_O, the minimum end-expiratory pressure (EPAP) was increased every 5 min by 4 cm H_2_O up to a maximum of 12 cm H_2_O, while maximum EPAP (14 cm H_2_O), maximum pressure support (10 cm H_2_O), and minimum pressure support (4 cm H_2_O) were kept stable. Individual maximum tolerable pressure support levels were then applied during right heart catheterization. In patients receiving oxygen therapy, the flow of oxygen was kept constant throughout the procedure.

### Statistical analysis

Data are shown as mean ± standard deviation (SD). For comparison of the two patient populations, Fisher’s exact test, Chi-square test and two-sided paired *t*-test were used as appropriate. Comparisons between baseline and intervention were made with two-sided paired *t*-test. Pearson correlation analysis was performed to assess changes in right and left sided filling pressures and changes in cardiac output. *P*-values <0.05 were considered statistically significant.

## Results

The study enrolled 33 patients, 21 with pre-capillary PH (PAH, *n* = 8; CTEPH, *n* = 13) and 12 with post-capillary PH associated with HFpEF. The demographics and baseline characteristics of the patients are shown in Table 1. None of the patients had a history of sleep-related breathing disorders. All patients were awake during the procedures and periodic breathing was not observed in any of these patients during the study.

**Table 1:** Patient characteristics at baseline

	All (*n* = 33)	Pre-capillary PH (*n* = 21)	Post-capillary PH (*n* = 12)	*p*-value*
Age (years)	68 ± 13	64 ± 15	72 ± 8	0.14
Female (%)	55	57	50	0.52
Body mass index (kg/m^2^)	28 ± 6	26 ± 5	31 ± 6	0.03
NYHA II/III/IV (*n*)	2/29/2	2/18/1	0/11/1	0.31
6 min walking distance (m)	315 ± 135	327 ± 147	289 ± 130	0.46
Right atrial pressure (mmHg)	12 ± 4	11 ± 4	13 ± 4	0.2
Mean pulmonary artery pressure (mmHg)	45 ± 11	46 ± 13	43 ± 9	0.46
Pulmonary arterial wedge pressure (mmHg)	13 ± 5	10 ± 3	18 ± 3	<0.001
Cardiac output (L/min)	4.2 ± 1.3	4.1 ± 1.1	4.4 ± 1.7	0.49
Cardiac index (L/min/m^2^)	2.2 ± 0.6	2.2 ± 0.4	2.3 ± 0.8	0.52
Stroke volume (ml)	63 ± 19	62 ± 20	64 ± 17	0.77
Pulmonary vascular resistance (dyn · s · cm^−5^)	660 ± 340	754 ± 367	495 ± 177	0.03
Peripheral oxygen saturation (%)	93 ± 3	93 ± 3	94 ± 2	0.95
Mixed venous oxygen saturation (%)	64 ± 6	63 ± 6	64 ± 6	0.21

*Pre-capillary versus post-capillary pulmonary hypertension

Compared to patients with pre-capillary PH, patients with post-capillary PH tended to be older and had a higher body mass index. The patients in both cohorts suffered from severe PH with an average PAPm of 46 and 43 mmHg, and a reduced cardiac index of 2.2 and 2.3 L/min/m^2^, respectively.

Familiarization with ASV on the day before right heart catheterization was associated with a significant drop of systolic blood pressure (prior to ASV therapy 131 ± 21 mmHg versus ASV therapy with maximum EPAP of 12 mmHg 122 ± 20 mmHg, *p* = 0.01) while diastolic blood pressure (73 ± 12 mmHg vs. 73 ± 12 mmHg, *p* = n.s.) and mean arterial blood pressure (90 ± 20 mmHg versus 89 ± 13 mmHg, *p* = n.s.) remained unchanged. Clinically, therapy initiation was uneventful and all patients tolerated the procedure without any side effects.

**Fig. 1:**
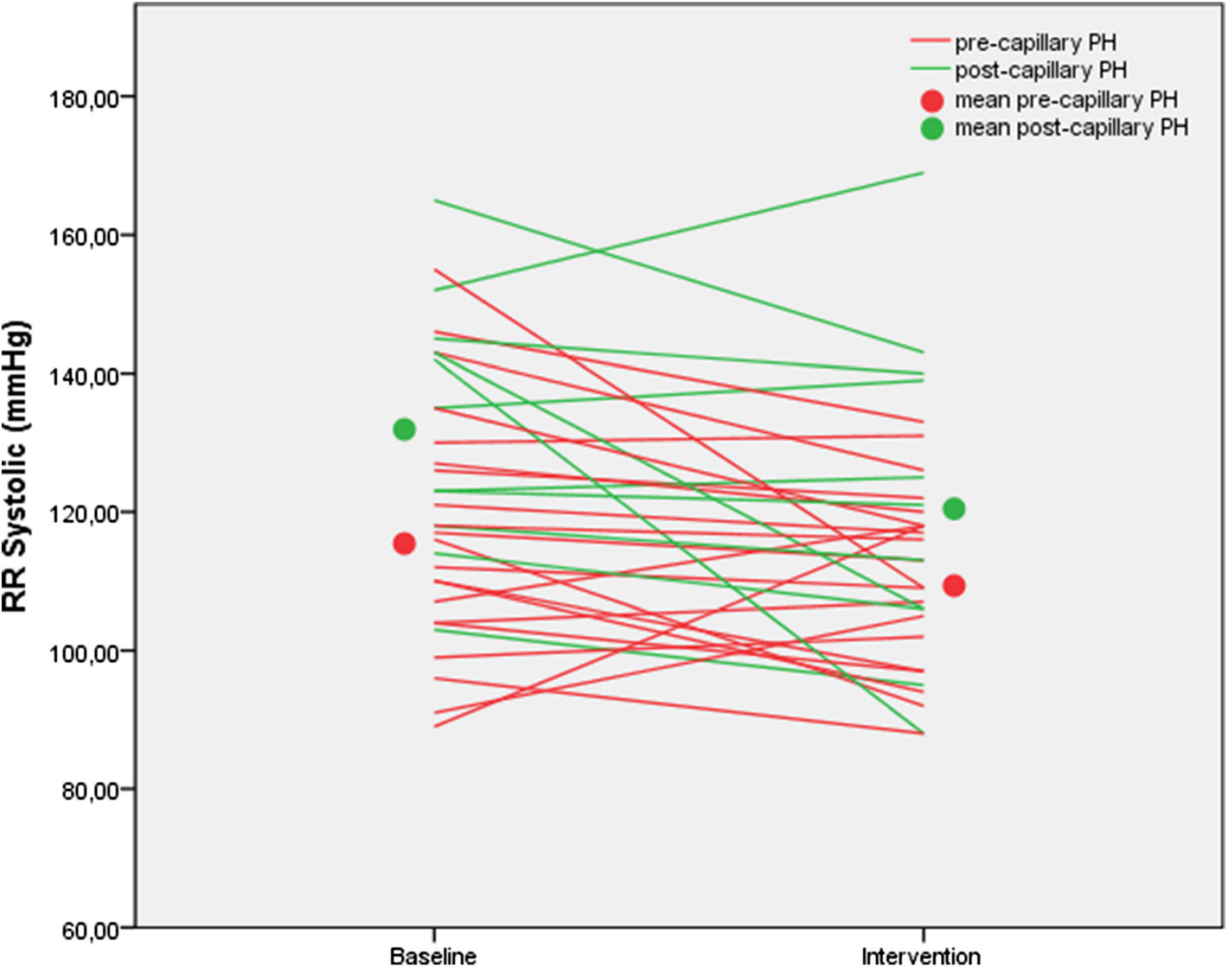
Systolic blood pressure at baseline and at the end of a 15-min period of adaptive servoventilation in patients with pre-capillary or post-capillary pulmonary hypertension. In the entire group, there was a significant decline in systolic blood pressure (RR systolic) by 8 mmHg (*p* = 0.01). In patients with pre-capillary pulmonary hypertension (PH), the systolic blood pressure dropped by 6 mmHg (*p* = 0.087); in patients with post-capillary PH, it dropped by 11 mmHg (*p* = 0.07)

**Fig. 2:**
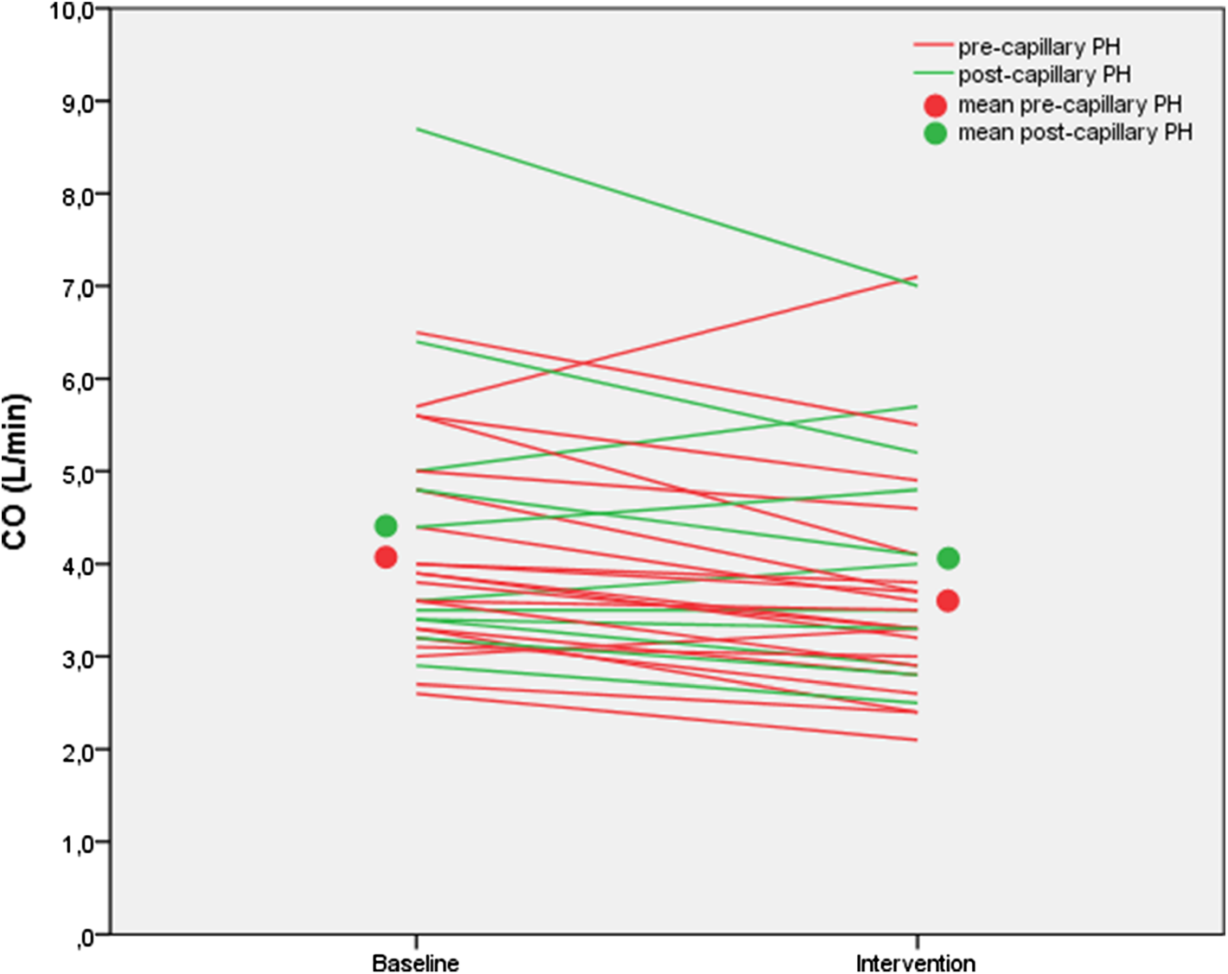
Cardiac output (CO) at baseline and at the end of a 15-min period of adaptive servoventilation in patients with pre-capillary or post-capillary pulmonary hypertension. In the entire group, cardiac output decreased by 0.4 L/min (*p* < 0.0001). In patients with pre-capillary pulmonary hypertension (PH), cardiac output decreased by 0.5 L/min (*p* = 0.001). In patients with post-capillary pulmonary hypertension (PH), cardiac output decreased non-significantly by 0.3 L/min (*p* = 0.11)

The individual maximum tolerable pressure settings were then applied the next day during right heart catheterization without causing adverse events. Twenty-nine patients (88 %) were ventilated at the highest-pressure settings (maximum EPAP 14 cmH_2_O, maximum pressure support 10 cmH_2_O). Hemodynamic effects have been summarized in Table 2. There was an average decline in systolic blood pressure by 8 mmHg (*p* = 0.01; Fig. 1) while the diastolic blood pressure didn’t change significantly (*p* = 0.40). The cardiac filling pressures increased (right atrial pressure +2.4 mmHg and PAWP + 3.6 mmHg; both *p*-values < 0.0001). PAPm decreased by 5 mmHg (*p* < 0.0001), PVR decreased by 10 % (68 dyn · s · cm^−5^; p = 0.013) and cardiac output decreased by 0.4 L/min (*p* < 0.0001). The individual changes in cardiac output are depicted in Fig. 2, which shows that the drop in cardiac output was less consistent in patients with post-capillary PH than in pre-capillary PH. The stroke volume did not change significantly (*p* = 0.41), while the heart rate declined by 7 beats per minute (*p* < 0.0001). There was no correlation between the change in PAWP and the change in cardiac output (*r* = 0.147, *p* = 0.414). In contrast, the change in cardiac output was inversely related to the change in right atrial pressure (*r* = −0.414, *p* = 0.017).

**Table 2:** Hemodynamic response to adaptive servoventilation in patients with pulmonary hypertension (whole group, *n* = 33)

	Baseline	Intervention	Recovery	p*
Systolic blood pressure (mmHg)	123 ± 19	115 ± 18	124 ± 19	0.010
Diastolic blood pressure (mmHg)	67 ± 12	66 ± 11	69 ± 12	0.40
Heart rate (min^−1^)	70 ± 13	64 ± 13	67 ± 12	<0.001
Right atrial pressure (mmHg)	11 ± 4	13 ± 4	11 ± 5	<0.001
Mean pulmonary artery pressure (mmHg)	45 ± 11	40 ± 11	44 ± 10	<0.001
Pulmonary arterial wedge pressure (mmHg)	13 ± 5	17 ± 5	14 ± 5	<0.001
Cardiac output (L/min)	4.2 ± 1.3	3.8 ± 1.2	4.2 ± 1.3	<0.001
Cardiac index (L/min/m^2^)	2.2 ± 0.6	2.0 ± 0.5	2.2 ± 0.5	<0.001
Stroke volume (ml/min)	63 ± 19	62 ± 20	64 ± 18	0.41
Pulmonary vascular resistance (dyn · s · cm^−5^)	660 ± 340	592 ± 353	644 ± 355	0.01
Peripheral oxygen saturation (%)	93 ± 3	96 ± 3	93 ± 4	<0.001
Mixed venous oxygen saturation (%)	64 ± 6	67 ± 6	62 ± 9	0.001
Mixed venous pCO_2_ (mmHg)	41 ± 5	38 ± 6	40 ± 7	<0.001

*Baseline versus intervention

The peripheral oxygen saturation increased by +4 % (*p* < 0.0001), as did the mixed venous oxygen saturation (+3 %; *p* = 0.0015). The mixed-venous pCO_2_ dropped by an average of 4 mmHg (*p* < 0.0001). After 15 min of recovery, all values had returned to baseline (Table 2).

The results were essentially the same for patients with pre-capillary and post-capillary PH, with the exception of the less consistent change in cardiac output as stated above (Tables 3 and 4).

**Table 3:** Hemodynamic response to adaptive servoventilation in patients with pre-capillary pulmonary hypertension (*n* = 21)

	Baseline	Intervention	Recovery	p*
Systolic blood pressure (mmHg)	117 ± 18	111 ± 13	119 ± 16	0.087
Diastolic blood pressure (mmHg)	65 ± 14	65 ± 9	68 ± 11	0.971
Heart rate (min^−1^)	69 ± 10	63 ± 11	66 ± 10	<0.001
Right atrial pressure (mmHg)	11 ± 4	13 ± 4	11 ± 5	0.002
Mean pulmonary artery pressure (mmHg)	46 ± 13	42 ± 12	44 ± 11	0.003
Pulmonary arterial wedge pressure (mmHg)	10 ± 3	15 ± 4	11 ± 3	<0.001
Cardiac output (l/min)	4.1 ± 1.1	3.6 ± 1.2	4.2 ± 1.3	0.001
Cardiac index (L/min/m^2^)	2.2 ± 0.4	1.9 ± 0.4	2.2 ± 0.4	<0.001
Stroke volume (ml/min)	62 ± 20	59 ± 20	63 ± 20	0.15
Pulmonary vascular resistance (dyn · s · cm^−5^)	754 ± 367	711 ± 372	750 ± 395	0.20
Peripheral oxygen saturation (%)	93 ± 2	97 ± 4	93 ± 3	<0.001
Mixed venous oxygen saturation (%)	65 ± 6	68 ± 6	62 ± 10	0.022
Mixed venous pCO_2_ (mmHg)	41 ± 5	37 ± 6	39 ± 8	0.001

*Baseline versus intervention

**Table 4:** Hemodynamic response to adaptive servoventilation in patients with post-capillary pulmonary hypertension (*n* = 12)

	Baseline	Intervention	Recovery	p*
Systolic blood pressure (mmHg)	133 ± 18	122 ± 23	133 ± 20	0.07
Diastolic blood pressure (mmHg)	71 ± 8	66 ± 13	69 ± 13	0.06
Heart rate (min^−1^)	73 ± 17	64 ± 17	68 ± 15	0.005
Right atrial pressure (mmHg)	13 ± 4	14 ± 4	13 ± 4	0.02
Mean pulmonary artery pressure (mmHg)	43 ± 9	37 ± 9	43 ± 9	<0.001
Pulmonary arterial wedge pressure (mmHg)	18 ± 3	21 ± 4	20 ± 2	0.16
Cardiac output (L/min)	4.4 ± 1.7	4.1 ± 1.4	4.3 ± 1.4	0.11
Cardiac index (L/min/m^2^)	2.3 ± 0.7	2.1 ± 0.6	2.3 ± 0.6	0.097
Stroke volume (ml/min)	64 ± 17	66 ± 19	65 ± 16	0.31
Pulmonary vascular resistance (dyn · s · cm^−5^)	495 ± 177	384 ± 190	458 ± 152	0.02
Peripheral oxygen saturation (%)	94 ± 2	95 ± 4	93 ± 4	0.29
Mixed venous oxygen saturation (%)	63 ± 6	65 ± 6	62 ± 7	0.02
Mixed venous pCO_2_ (mmHg)	42 ± 4	38 ± 6	41 ± 5	0.01

*Baseline versus intervention

## Discussion

Our data show that ASV, at least when administered at relatively high pressure levels, has hemodynamic consequences in patients with PH including an increase in filling pressures, and declines in systolic blood pressure, PAPm, PVR, and cardiac output.

The slight increases in filling pressures (RAP +2 mmHg, PAWP +4 mmHg) were probably caused directly by the increased intrathoracic pressure during ASV, and would not be expected to be clinically relevant. However, our data also show that right heart catheterization performed during noninvasive ventilation may result in over interpretation of the PAWP and could therefore lead to a misclassification of pre-capillary as post-capillary PH, at least in some patients.

There was a considerable decline in PAPm and PVR. The latter was caused by a drop in PAPm (−5 mmHg) and an increase in PAWP (+4 mmHg). The drop in PAPm was substantial and cannot be directly explained by increased intrathoracic pressures during ASV, as this would have caused the PAPm to increase as well. The drop in cardiac output can explain part of the observed effect on PAPm to some extent, although not entirely. Our data show that ASV was associated with slight improvements in peripheral and mixed venous oxygenation as well as a fall in the mixed venous pCO_2_, and it is possible that these effects resulted in some pulmonary vasodilation.

Perhaps the potentially most relevant effects seen in our study were the declines in systolic blood pressure and cardiac output. It is not entirely clear why only the systolic blood pressure decreased while the diastolic blood pressure remained unchanged, especially as the drop in cardiac output was not caused by a decline in stroke volume but almost entirely by a drop in heart rate. However, the observed effects on blood pressure and heart rate were consistent with previous studies on continuous positive airway pressure or NIV in patients with left heart disease [12–15]*.* These effects may be explained, at least partly, by reduced cardiac sympathetic nerve activity or increased parasympathetic nerve activity, which may result from stimulation of pulmonary stretch receptors due to lung inflation [15, 16].

Nevertheless, the decline in cardiac output was a somewhat unexpected finding, which has been described with the administration of high pressure during NIV in healthy individuals [17, 18] and in patients with chronic obstructive pulmonary disease [19], but not in most studies addressing the use of NIV patients with left heart disease [7, 8, 11]. One exception was a study by Philip-Joet et al. that reported a decrease in cardiac output during administration of NIV in patients with left heart disease and a low PAWP [20]. This observation, however, is not fully in line with the results of our study as we did observe a decline in cardiac output in some patients with a PAWP >15 mmHg, albeit less consistent than in patients with pre-capillary PH. Hence, the effects of ASV on cardiac output seem to be less predictable in patients with post-capillary PH than in patients with pre-capillary PH, were a drop in cardiac output was a very consistent finding. The changes in cardiac output did not correlate with the changes in PAWP but there was an inverse correlation between the changes in cardiac output and the changes in right atrial pressure, possibly indicating additional strain on the right ventricle induced by ASV.

ASV was well tolerated in all of our patients, both at the familiarization day as well as during right heart catheterization. There were no side effects and none of the patients reported any discomfort, except for what was caused by the facemask. Of note, all of our patients were clinically and hemodynamically stable. We cannot conclude from our data that ASV would also be safe if applied to patients with severe PH, right-sided heart failure and a low blood pressure. On the first glimpse, the drop in PAPm seems re-assuring. However, our data raise some concerns as any drop in blood pressure, cardiac output, or both in unstable PH patients could lead to hemodynamic decompensation and even death due to right heart failure. Such patients need to be studied further but our results at least raise the possibility that non-invasive ventilation may have deleterious hemodynamic consequences in patients with advanced right heart failure.

Recently a multicenter, randomized controlled trial on ASV therapy in patients with heart failure with reduced left ventricular ejection fraction failed to show a statistically significant difference between patients randomized to ASV therapy and those in the control group in the primary endpoint of time to all-cause mortality or unplanned hospitalization for worsening heart failure. There was, however, a statistically significant increased risk in all-cause and cardiovascular mortality in patients receiving ASV therapy [21]. Whether our results, exemplifying potentially adverse hemodynamic effects of ASV in patients with pre- or post-capillary PH can be transferred to this patient group and may help to explain these results remains to be elucidated.

Our study has several strengths and limitations. Strengths include the inclusion of well-characterized patients with severe pre-capillary and post-capillary PH, the familiarization with ASV prior to right heart catheterization and the fact that all patients had invasive hemodynamic measurements. Limitations included the single-center setting, the relatively small number of patients, the application of only one form of NIV, i.e. ASV, and the limited duration of NIV. Different modes and longer applications of NIV may result in different results but we aimed to study the acute effects on NIV in these patients and we wanted to make sure not to extend the invasive procedure any longer than necessary. The pressure support used in our study was relatively high compared to other studies [17, 18] and our results may not be fully applicable to lower pressure settings. In addition, day-time measurements in awake patients may not necessarily provide the same results as night-time measurements in asleep patients.

In conclusion, our data showed that administration of NIV in stable patients with pre-capillary or post-capillary PH resulted in small but consistent declines in heart rate, systolic blood pressure, PAPm, PVR and cardiac output. These hemodynamic effects were clinically unapparent in the patients under study, but our findings suggest that NIV should be used with caution in PH patients presenting with hypotension and low cardiac output failure.

## Abbreviations

ASV: Adaptive servoventilation
PH: Pulmonary hypertension
RAP: Right atrial pressure
PAWP: Pulmonary arterial wedge pressure
PAPm: Mean pulmonary artery pressure
PVR: Pulmonary vascular resistance
CTEPH: Chronic thromboembolic pulmonary hypertension
HFpEF: Heart failure with preserved ejection fraction
NIV: Noninvasive ventilation
CPAP: Continuous positive airway pressure
EPAP: The end-expiratory pressure
